# Melatonin for the Management of Preeclampsia: A Review

**DOI:** 10.3390/antiox10030376

**Published:** 2021-03-03

**Authors:** Annie Langston-Cox, Sarah A. Marshall, Daisy Lu, Kirsten R. Palmer, Euan M. Wallace

**Affiliations:** 1 The Ritchie Centre, Department of Obstetrics and Gynecology, School of Clinical Sciences, Monash University, 246 Clayton Road, Clayton, VIC 3168, Australia; annie.cox1@monash.edu (A.L.-C.); sarah.marshall@monash.edu (S.A.M.); dlu28@student.monash.edu (D.L.); kirsten.palmer@monash.edu (K.R.P.); 2 Monash Health, Clayton, VIC 3168, Australia

**Keywords:** preeclampsia, melatonin, antioxidant, placental biology, mitochondrial function

## Abstract

Preeclampsia is a disease specific to pregnancy characterised by new-onset hypertension with maternal organ dysfunction and/or fetal growth restriction. It remains a major cause of maternal and perinatal morbidity and mortality. For sixty years, antihypertensives have been the mainstay of treating preeclampsia and only recently have insights into the pathogenesis of the disease opened new avenues for novel therapies. Melatonin is one such option, an endogenous and safe antioxidant, that may improve the maternal condition in preeclampsia while protecting the fetus from a hostile intrauterine environment. Here we review the evidence for melatonin as a possible adjuvant therapy for preeclampsia, including in vitro evidence supporting a role for melatonin in protecting the human placenta, preclinical models, vascular studies, and clinical studies in hypertension and pregnancy.

## 1. Introduction

Preeclampsia is a systemic maternal-fetal disorder specific to human pregnancy. It is characterised by new-onset hypertension after twenty weeks gestation and other end-organ damage, such as renal or liver impairment, haematological involvement, neurological excitability and/or fetal growth restriction. Preeclampsia is the leading cause of preterm delivery and is often accompanied by fetal compromise, in particular impaired fetal growth [1]. In addition to this, the knowledge that underlying preeclampsia is a dysfunctional placenta is why fetal growth restriction is increasingly included in definitions of preeclampsia [1].

Regarding placental dysfunction, impaired placental perfusion causes chronic and worsening hypoxic-reperfusion injury to the placenta across pregnancy. This injury underlies the excessive release of antiangiogenic factors into the maternal circulation [2,3]. In turn, these factors cause widespread maternal endothelial dysfunction that leads progressively to increased maternal systemic vascular resistance and activation of the maternal coagulation and immune systems [4,5]. If left untreated, this progressive vascular dysfunction leads to dangerously high blood pressures and stroke, systemic organ failure, and cerebral oedema and seizures. While many women who develop preeclampsia, particularly in the setting of late-onset disease, will have good outcomes, this condition remains the leading cause of maternal death and morbidity and a major cause of preterm birth that accounts for significant perinatal mortality and mortality worldwide [6,7,8]. Globally, preeclampsia accounts for nearly 75,000 maternal deaths and 700,000 neonatal deaths annually. Even in high-resource settings, preeclampsia accounts for upward of 15% of maternal deaths [6,7,8]. After delivery, the burden of disease for preeclamptic mothers continues. Women who have suffered preeclampsia during their pregnancy have an increased chance of developing cardiovascular compromise and ongoing related morbidity throughout their lives. This is likely due to, at least in part, residual endothelial dysfunction from significant vascular stress during the pregnancy period [9,10]. Similarly, the effects of preeclampsia on the fetus may be much more pronounced than those attributed to preterm delivery and growth restriction. Epigenetic priming means that babies born to preeclamptic mothers are at an increased risk of a host of metabolic conditions throughout their lives [11,12]. So, while generally considered a maternal disease, preeclampsia at its core is a placental disease that imposes disease burden on both mother and fetus. Therapies targeting preeclampsia need to consider both patients.

In that regard, the management of preeclampsia depends on the gestation at onset, the severity—both maternal and fetal—and the rate of progression [1]. However, the ultimate treatment of preeclampsia is removal of the offending organ—the placenta. This is why preeclampsia remains a leading cause of prematurity because delivery of the placenta requires, of course, delivery of the fetus. Timing delivery is then a balance between the interests of the mother and the interests of the fetus. The interests of the woman with preeclampsia are always best served by delivery. Delivery prevents worsening hypertension and thereby avoids related complications including stroke, liver failure and kidney failure. However, early delivery may not best serve the baby, particularly if very preterm. Beyond neonatal demise, preterm delivery is a risk factor for a host of conditions that will affect a baby well into their adult life, including cerebral palsy, visual and hearing problems, respiratory difficulties, cardiovascular compromise, renal impairment, and learning and behavioural problems [13]. Even late preterm delivery (<37 weeks) increases the risk of lifelong cardiovascular and renal complications [14,15]. In this way, iatrogenic premature birth, though often necessary to save the life of the mother, also may come at significant cost to the baby [13]. So, at very early gestations, to offset those fetal risks, the maternal health risks are mitigated by controlling blood pressure with antihypertensives. This allows safer (for the mother) prolongation of the pregnancy to improve fetal maturity and reduce risks of neonatal mortality and morbidity [16].

Thus, antihypertensive treatment manages the high blood pressure to reduce maternal risks, particularly of stroke. This has been very successful. The use of antihypertensives in women with preeclampsia has greatly reduced rates of both maternal morbidity and mortality worldwide. However, antihypertensive treatment does not treat the underlying disease. Nor does it slow disease progression, although there is some debate about that. Its effect is very much limited to managing high blood pressure to reduce attendant maternal risks. That is not to say that antihypertensive treatment is without complication for both mother and baby, and the antihypertensive of choice remains contentious. Indeed, improvements over recent decades in maternal and perinatal outcomes in pregnancies complicated by preeclampsia have come mostly from the ability to deliver earlier than was previously possible, safe in the knowledge that better neonatal care has resulted in better outcomes for the preterm infant. Antenatal corticosteroids aside, there have been no real advances in the obstetric care of the woman with preeclampsia since the introduction of antihypertensives in the 1950s. Pharmacologically, the management of preeclampsia has been largely “treading water” [17].

However, this may be all set to change. Recent insights into the mechanisms underlying the maternal aspects of preeclampsia have offered the long-awaited promise of new treatments [17]. In particular, the recognition that maternal endothelial dysfunction due to placental vasoactive peptides [2,18] is a central feature of the disease has offered the promise of new, targeted therapies that might address the central causes of the hypertension rather than the hypertension per se [16,17]. Pathogenic mechanistic insights have led to the recognition that we must look to therapeutic approaches beyond antihypertensives if we wish to tackle the underlying disease processes that drive preeclampsia. Such developments have revolutionised research for preeclampsia and, for the first time in sixty years, soon we may be able to offer novel medical management to pregnant women with preeclampsia and their babies.

## 2. The Search for Novel Therapies

**Figure 1:**
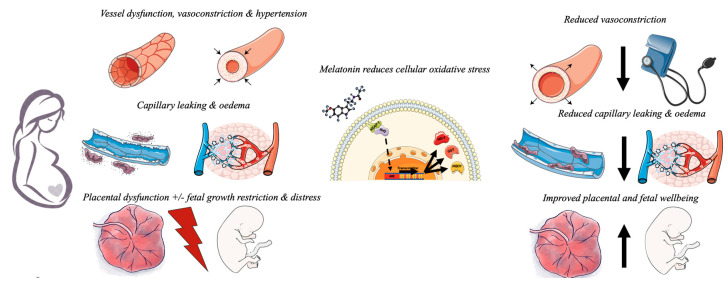
Preeclampsia involves widespread vascular dysfunction resulting in peripheral vasoconstriction which manifests as maternal hypertension. Capillary leakage results in oedema and impaired placental oxygenation results in placental dysfunction and fetal distress with, or without, fetal growth restriction. Melatonin is an antioxidant that reduces oxidative stress within the cells of the placenta and vasculature. Melatonin releases Nrf2 from intracellular binding by KEAP-1 allowing it to translocate to the nucleus of the cell. Here, Nrf2 activates the antioxidant response element of “safeguarding genes” resulting in transcription and translation of a number of antioxidant proteins. These proteins induce redox reactions to neutralise excessive intracellular reactive oxygen species that would otherwise cause damage to DNA and protein production essential for cell function. This allows melatonin to improve vascular function reducing vasoconstriction, reduce capillary leakage and improve placental function. Melatonin may also directly protect the developing fetal brain.

In 1989, Jim Roberts and colleagues suggested that preeclampsia might be due to widespread maternal endothelial dysfunction [19]. For the first time, a mechanism that might explain the majority of the clinical features of the syndrome, not just the hypertension, had been proposed. In essence, Roberts suggested that disturbed vascular function, including altered tone and permeability, was the cause of hypertension, peripheral and cerebral oedema, and proteinuria [19,20]. The other features of preeclampsia such as liver injury, renal injury, thrombocytopenia and, ultimately, eclampsia itself were also thought to reflect progressive endothelial dysfunction across diverse target organs [19] (Figure 1). While our understanding of preeclampsia has evolved somewhat since then, Roberts’ hypothesis was important because the recognition that endothelial dysfunction was a key mechanism underlying preeclampsia triggered the need to identify the cause(s) of that endothelial disturbance. It gave rise to the concept that preeclampsia is a two-step process: impaired placentation leading to progressive placental ischemia-reperfusion and oxidative injury, in turn causing the excessive release of vasoactive factors into the maternal circulation that induced endothelial dysfunction [4]. While not explaining all aspects of preeclampsia, it remains generally accepted that the maternal syndrome of preeclampsia is due, by and large, to widespread vascular endothelial dysfunction [20], and that the endothelial dysfunction was caused by substances released by a chronically injured placenta. The next significant advance was the identification of the candidate vasoactive substances that were causing the dysfunction. Three antiangiogenic agents have been proposed as major contributors—the soluble splice variant of the fms-like tyrosine kinase receptor-1 (sFlt1), the soluble cleavage product of the transforming growth factor (TGF-β1) coreceptor endoglin (sEng) and the proinflammatory cytokine member of the TGF-β super family activin A [2,5,21,22,23].

Circulating levels of both sFlt1 and sEng are many fold higher in women with preeclampsia than in those with a healthy pregnancy and levels correlate with disease severity [22,24]. Further, experimentally the administration of either, or both, sFlt1 and sEng to rodents induces many features of human preeclampsia including maternal hypertension, proteinuria, glomerular endotheliosis, thrombocytopenia, and elevated liver enzymes [22,25,26,27], as does activin [23]. Removal of sFlt1 by plasmapheresis also temporarily moderates the severity of hypertension in women with preeclampsia [28]. Hypoxic insult induces post-transcriptional alternate splicing of mRNA for the membrane receptor Flt-1, to lose the transmembrane and intracellular signaling components of Flt-1 while preserving the extracellular ligand binding site [29]. In contrast, hypoxia directly triggers post-translational cleavage of the endoglin protein membrane receptor into its soluble form [22], which competitively binds TGF-β1. It is thought that sFlt1 and sEng induce endothelial dysfunction by either sequestering or antagonising pro-angiogenic factors that are vital for normal endothelial health such as vascular endothelial growth factor (VEGF) and placental growth factor (PlGF) and TGF-β respectively. These three key proangiogenic compounds are essential for maintaining blood vessel integrity by inducing phosphorylation, and hence activation of nitric oxide (NO), a potent vasodilator essential in facilitating endothelial relaxation [30]. When competitive binding by sFlt-1 and sEng makes VEGF, PlGF and TGF-β1 unavailable, progressively worsening vascular dysfunction characteristic of preeclampsia ensues.

Similarly, circulating maternal levels of activin are 10–20-fold higher in women with preeclampsia compared to those with a healthy pregnancy, secondary to increased placental production driven by oxidative stress [31,32,33]. Much like sFlt and sEng, activin is antiangiogenic [34] and has been shown to inhibit endothelial proliferation and disrupt endothelial integrity in vitro [35,36]. Activin A binds to activin receptor II (ARII), which combines with type-1 activin receptor like kinase-4 (ALK-4) resulting in phosphorylation and nuclear translocation of post-receptor transcription factor Smad2/3 [23,36,37]. Excess activation of this pathway induces NADPH Oxidase 2 (Nox2) signaling and results in cellular accumulation of superoxide species and adhesive molecules. As with sFlt and sEng, the increased levels of activin in a preeclamptic woman lead to further vascular dysfunction, permeability and oedema, exacerbating the clinical syndrome of preeclampsia [23,31,38,39]. Activin also stimulates the release of endothelin, a potent vasoconstrictor, from the endothelium [40], consistent with it being able to cause hypertension. As would be required for activin to have direct effects on the endothelium, endothelial cells express both type I and II activin receptors and in late pregnancy activin itself can be immunolocalized to both the maternal and fetal vascular endothelium [41,42]. The likely cause of these increased activin levels in preeclampsia is placental oxidative stress, a key feature of the disease [23,32]. Of course, increased levels of activin in women with preeclampsia and possible placental mechanisms underlying those increased levels do not, by themselves, tease apart cause and effect. However, circumstantial evidence that excess circulating activin may indeed have a causative role is offered by the observation that in women who subsequently develop preeclampsia levels of activin are significantly increased as early as 8–13 weeks of pregnancy, many months before the clinical onset of hypertension [43]. It is also intriguing that levels of activin are increased in women with gestational diabetes, a condition with an increased incidence of preeclampsia [44]. In short, the result of the imbalance between these three antiangiogenic and pro-angiogenic factors such as PlGF and follistatin is increased endothelial oxidative stress—the likely final pathway underlying the systemic maternal endothelial dysfunction.

Not surprisingly, attention has now turned to therapies that might prevent the release of sFlt1, sEng or activin A, or perhaps more importantly, antagonize their antiangiogenic effects. Given that oxidative stress both increases the release of these antiangiogenic factors [32,40,45,46] and is a major mechanism by which they exert their damaging effects on vascular endothelium suggests that targeting oxidative stress, both within the placenta and in the maternal endothelium, may be an effective therapeutic approach [47,48,49]. In this regard, melatonin, an endogenous hormone, known to be safe in pregnancy and with potent antioxidant capacity is a promising agent [50].

## 3. Melatonin in Normal Pregnancy and Preeclampsia

Melatonin (5-methoxy-N-acetyltryptamine) is produced primarily by the pineal gland, providing circadian and seasonal timing cues. It is synthesized from serotonin through sequential acetyl transferase, to form *N*-acetylserotonin, and methylation to form melatonin [51]. In addition to cueing the body clock, melatonin is also a powerful antioxidant, acting both directly as a highly effective scavenger of reactive oxygen and nitrogen species itself [52] and indirectly by stimulating a cassette of endogenous antioxidant enzymes including, but not limited to, glutathione peroxidase, glutathione reductase, superoxide dismutase, and catalase [52,53].

In human pregnancy, night time, but not daytime, maternal melatonin levels increase with advancing gestation, falling again postpartum [52]. The increasing levels at the end of pregnancy are thought to be important in the diurnal “training” of fetal physiology and behaviour. This sequential rise in melatonin across pregnancy may also play a role in stimulating labour, and melatonin has been proposed as a therapeutic adjuvant for induction of labour [54]. Intriguingly, night time levels of melatonin are lower in women with severe preeclampsia than in those with a healthy pregnancy [55]. In fact, the degree of night time melatonin deficiency correlates with preeclampsia disease severity [56]. The majority of circulating melatonin in pregnancy is thought to be of maternal pineal origin [51], much as it is outside of pregnancy. However, recent studies have identified that the expression of the two melatonin-synthesizing enzymes, aralkylamine *N*-acetyltransferase and hydroxyindole O-methyltransferase, and the two melatonin receptors, MT_1_ and MT_2_, are reduced in the preeclamptic placenta compared to healthy placentae from normotensive women [51]. As such, impaired placental production may underlie the reduced maternal levels of melatonin in women with preeclampsia and the normal biological effects of melatonin within the placenta may be reduced in preeclampsia. It is plausible that impaired melatonin activity, and therefore impaired endogenous antioxidant defenses, in the preeclamptic placenta contributes to the oxidative stress central to this disease. As such administration of melatonin to women with preeclampsia may reduce placental oxidative stress, reduce the production of sFlt1, sEng and activin, and improve placental function. Melatonin may also improve maternal endothelial function both directly, via endothelial melatonin receptors [57], and indirectly by reducing circulating levels of sFlt1, sEng, and activin A.

The antioxidant effects of melatonin have historically been attributed to activation of nuclear factor erythroid-like factor-2 (Nrf2), an endogenous inducer of cellular antioxidants. During homeostatic conditions the Nrf2 protein is bound to Kelch-like ECH associated protein 1 (KEAP-1) within the cytosol of the cell [58]. This process prevents proteosomal degradation of Nrf2 to ensure abundant Nrf2 remains ready to translocate to the nucleus of the cell during times of increased cellular oxidative stress. Oxidative stress triggers ubiquitination of KEAP-1 through directly modifying cysteine components of the protein structure, releasing Nrf2 into the cytosol of the cell [59]. In the nucleus Nrf2 binds small maf-proteins in the promotor region of the antioxidant response element of so-called safeguarding genes within the cell nucleus [59]. This stimulates increased transcription and translation of a host of antioxidant enzymes and phase two enzymes, namely NADPH dehydrogenase (quinone) 1 and glutathione S-transferase and the antioxidant heme oxygenase-1 [59]. These enzymes then undergo a series of redox reactions within the cytosol of the cell to neutralise, or “scavenge”, damaging oxygen free radicals [60]. When the amount of damaging reactive oxidative species (ROS) overwhelm the capacity of this inbuilt antioxidant rescue mechanism, oxygen free radicals directly alter protein structure and damage cellular DNA, producing abnormal spliced proteins such as sEng and sFlt-1 and triggering inflammatory cascades to release cytokines such as activin A [60]. For good reason, stimulators of the Nrf2 pathway, such as resveratrol and sulforaphane, have received much attention for their impressive antioxidant capacity, and ability to both maintain endothelial health [49,61,62,63] and protect the placenta [48,49,64]. In fact, further evidence supports a role for the KEAP-1-Nrf2 pathway in protecting the fetus against complications from epigenetic priming that arise from a pregnancy overwhelmed by oxidative stress [11,12,65].

Not surprisingly given its antioxidant properties, melatonin has been shown to reduce placental production of antiangiogenic compounds from term placentae in vitro [47,66,67]. Melatonin also reduces trophoblastic debris from early trimester placentae exposed to preeclamptic serum [68]. In placental explants, melatonin reduced markers of oxidative stress induced by the superoxide generator xanthine/xanthine oxidase and increased production of Nrf2 and the downstream antioxidant enzyme heme oxygenase-1, suggesting the Nrf2 pathway was, at least in part, responsible for this effect [47]. Although melatonin did not alter cell production of markers of endothelial activation in endothelial cells, it did prevent disruption to the cell monolayer [47]. In isolated trophoblast cells, melatonin significantly decreased secretion of sFlt-1 [67]. While these studies did not identify a reduction in antiangiogenic compounds from placental explant tissue, the dose of melatonin used was equivalent to that in cell culture experiments. It is likely that higher doses are needed to penetrate explant tissue and induce a measurable effect. Further evidence of the endothelial protective capacity of melatonin was identified when melatonin prevented a rise in intracellular cell adhesion molecule-1 (ICAM-1) from endothelial cells exposed to trophoblast debris serum [66]. Melatonin also prevented a rise in nitrotyrosine in these placental explants exposed to preeclamptic serum [66].

An additional pathway by which melatonin mitigates placental oxidative stress may well begin in the organelles most reliant on oxygen supply and thus most affected by hypoxia: the mitochondria [69,70]. A role for disturbed mitochondrial dysfunction in preeclampsia was first recognised in the 1990s [71]. Mitochondrial dysfunction after hypoxic reperfusion injury is now accepted as the driver for ROS build-up in severe disease [72,73,74,75,76]. Mitochondria are responsible for multiple functions, including respiration and production of cellular energy, homeostatic regulation of ROS and the intrinsic pathway of apoptosis. Mitochondria form a dynamic network within the cell and constantly undergo repeated fission and fusion events whereby multiple mitochondria fuse to a large single mitochondria (fusion) or split into multiple smaller mitochondria (fission) [77,78,79]. The balance of this process is essential for cellular homeostasis and ensures maintenance of healthy mitochondrial structure with stability of the matrix membrane which hosts the five complexes of the electron transport chain [78]. Low oxygen tensions, as in preeclampsia, induce abnormal fission and fusion dynamics, generating small mitochondria with low motility [80]. Hypoxic insult induces mitochondrial permeabilization and breakdown and, when extensive, can initiate pathways of mitophagy and intrinsic cellular apoptosis [75]. The mitochondrial electron transport chain, essential for production of ATP for cellular energy, is reliant on the presence of oxygen for oxidative phosphorylation of ADP into ATP [81]. During respiration, electrons move through a series of complexes, allowing the formation of a proton gradient in the intermembrane space which then allows passive diffusion of hydrogen ions through the final complex ATP synthase where ADP is phosphorylated into ATP [80,81]. Without oxygen to act as an electron accepter, as occurs in an oxygen starved-preeclamptic placenta, electrons are unable to flow along the electron transport chain and instead leak into the matrix space [82]. Here, electrons react with oxygen to form charged superoxide species: the damaging ROS of preeclampsia. To a degree, this process occurs even in the presence of oxygen to allow homeostatic regulation of the ROS necessary for physiological cellular processes [82]. However, when ROS formation exceeds antioxidant enzyme production, the electron transport chain becomes a source of the very ROS that drive placental antiangiogenic protein formation in preeclampsia [82]. Indeed, mitochondrial function is disturbed in preeclampsia [71,72,83] suggesting that targeting the mitochondria offers an attractive therapeutic option to reduce oxidative stress in this disease. Again, melatonin appears a suitable candidate for that therapy [70,84,85].

Melatonin is highly expressed in the mitochondria of placental trophoblasts [86,87] and is responsible for the production of key antioxidant enzymes in this organelle [70]. Indeed, melatonin is known to protect mitochondrial function [88], reduce electron leakage and ameliorate ROS production of the electron transport chain [89,90], particularly in the face of hypoxic-reperfusion injury [84,91,92]. In the placenta of obese women, placentae characterised by trophoblastic ROS production similar to that of preeclampsia, melatonin significantly improves the function of the electron transport chain [93]. Specifically, in the term placental syncytiotrophoblast of obese women, melatonin improves the spare respiratory capacity, a marker of cellular reserve, an important feature for placentae exposed to oxygen deficient environments [93]. Melatonin also increases the maximal respiration and correspondingly reduces the placental production of superoxides. These findings indicate that melatonin may act as a protective buffer for the placental mitochondrial electron transport chain against the damaging effects of hypoxic-ischaemic reperfusion injury and the resultant superoxides. Whether melatonin exerts this action via antioxidant effects, such as activation of the Nrf2 antioxidant response element pathway, or by directly modulating the components of the electron transport chain remains unclear. This is certainly worthy of study. However, a similar compound to melatonin, sulforaphane, has action at the level of the mitochondria and this appears to be via potent Nrf2 antioxidant activity as well as by modulating mitochondrial fission and fusion and the complexes of the electron transport chain [94]. It would be worth investigating the capacity and mechanisms of action of melatonin in improving mitochondrial function in preeclamptic placentae or suitable in vitro hypoxic-reperfusion models of injury.

In addition to its beneficial effects in the placenta, melatonin also has reparative actions on the endothelium. Studies of melatonin on vascular cells have primarily focussed on human umbilical vein endothelial cells (HUVECs). In these cells, a microarray analysis demonstrated that melatonin significantly modulates expression of genes involved in apoptosis, cell differentiation and proliferation [95]. In an in vitro hypoxia-reoxygenation model in endothelial cells, melatonin treatment prevented hypoxic—reperfusion injury by preventing a rise in ROS and corresponding impaired cell migration and proliferation in a dose-dependent manner, without negatively affecting cell viability [96]. More recently, melatonin has been shown to have anti-ROS activity in toxic environments of oxidative stress and hypoxia, with reduced endothelial cell proliferation and tube formation [97]. These studies provide supporting evidence that melatonin displays antiangiogenic effects by suppressing the proliferation of endothelial cells, an effect achieved by the downregulation of hypoxia inducible factor 1α (HIF-1α), ROS and vascular endothelial growth factor. Suppressing these pathways is an important step in ameliorating the excessive production of the sEng and sFlt-1 seen in the preeclamptic placenta.

Though melatonin certainly freely crosses the placenta, this is not a concern. In fact, melatonin has also showed promise for the management of fetal growth restriction (FGR), a condition that goes hand in hand with preeclampsia and can be, broadly, viewed as the fetal manifestation of placental insufficiency, much as preeclampsia is the maternal manifestation. Evidence from animal models of impaired placentation and FGR support a role for melatonin in improving placental function and fetal outcomes. For example, following early pregnancy nutritional restriction in the sheep, oral maternal administration of melatonin improved uteroplacental blood flow—both uterine and umbilical—and fetal weight [98]. Melatonin is also able to protect the fetal brain and normalize early neurodevelopment in a fetal sheep model of FGR using umbilical cord occlusion [99]. Improved fetal growth was also afforded by maternal melatonin in both a nutritionally restricted rat model of FGR [100] and in a rat model where FGR was imposed by transient occlusion of the utero-ovarian arteries in mid-pregnancy [92]. In each of these models the administration of melatonin was associated with decreased placental oxidative stress and increased antioxidant enzymes [65,98,100]. Studies of melatonin for FGR have shown that oral melatonin taken by women during pregnancy crosses the placenta into the fetal circulation where it may protect the fetal brain from the harmful effects of oxidative stress [101]. In a pilot FGR trial where 8 mg melatonin per day was given to 12 women with severe FGR, a 200-fold increase in both maternal and fetal melatonin levels without maternal or fetal adverse effects was seen [102]. The clinical utility of melatonin as a neuroprotective therapy to improve neonatal outcomes in the setting of FGR is under ongoing investigation [103]. This neuroprotective feature is an appealing feature of melatonin for conditions of placental oxidative stress such as FGR and preeclampsia, killing two birds with one stone as it were.

In summary, melatonin has beneficial effects both within the placenta and on the maternal endothelium. It improves endothelial cell health by reducing inflammatory activation and antiangiogenic factor secretion [66], most likely through improved mitochondrial function, and increases expression of antioxidant enzymes in trophoblasts [67]. So what is the evidence for melatonin as an antihypertensive?

## 4. Melatonin as an Antihypertensive

Melatonin is an antihypertensive, reducing blood pressure in experimental animal models of hypertension, in healthy individuals, and in patients with established hypertension [7,57,104,105,106]. In fact in early animal studies, removal of the pineal gland, responsible for endogenous melatonin production, resulted in hypertension [107,108] with exogenous administration of melatonin reversing this vasoactive effect [109]. These initial studies provided evidence that melatonin was involved in cardiovascular regulation and prompted further investigation. Since then, a number of studies have identified that exogenous melatonin modifies blood flow, with effects varying depending on the vasculature.

The mechanisms underlying melatonin’s antihypertensive actions remain to be fully elucidated [57], but are likely to involve some or all of: central effects, systemic anti-inflammatory, antioxidant, and lipid lowering effects, direct effects on the myocardium and direct effects on the vascular endothelium [57]. In pregnancy melatonin increases umbilical blood flow in sheep [110], uterine artery blood flow in cows [111] and decreases cerebral blood flow in young rats [112]. These observations are consistent with improved placental function. Interestingly, in a chronic nitric oxide (NO) inhibited rat model of hypertension, melatonin treatment for 5 days significantly reduced basal mean arterial pressure [113]. A chronic intermittent hypoxic rat model induced endothelial dysfunction in the aorta by decreasing relaxation when exposed to the endothelium-dependent vasodilator acetylcholine [114]. This was mitigated by melatonin. Such findings were attributed to an increase in NO availability and increased protein expression of endothelial NO synthase (eNOS) in the aorta [114]. In this study, melatonin also decreased mRNA expression in the aorta of endothelial dysfunction markers including vascular cell adhesion molecule-1 (VCAM-1), ICAM-1 and E-selectin. While useful in understanding the effect of melatonin on the cardiovascular system, none of these animal models were models of preeclampsia. Melatonin is believed to directly activate receptors located on endothelial and vascular smooth muscle cells, and through its antioxidant properties, indirectly modulate vascular tone [57]. In the vasculature, melatonin receptors have conflicting effects, inducing vasoconstriction via the receptor MT_1_ and vasodilation via MT_2_, as first demonstrated in isolated rat caudal arteries [115]. It is the relative distribution differences of the melatonin receptors that elicits differential vascular responses in different blood vessels, and often selectively potentiates the vasoconstrictor response to serotonin. For example, in pigs, melatonin appears to reduce vasoconstriction in the coronary artery and increase vasoconstriction in the pulmonary artery, while vasoconstricting the rat coronary artery [116]. In contrast, melatonin induces vasodilation in rabbit aorta, iliac and renal vasculature [117,118] and the rat aorta [119]. Conversely, melatonin increases vasoconstriction in the coronary artery of pigs, but only if serotonin is present [120]. With such variability, the studies of the vascular actions of melatonin have revealed a substantial heterogeneity of effects.

Only recently were the mechanisms behind melatonin-induced vasodilation explored. Now, we understand that melatonin can affect arterial blood pressure and blood flow to tissues and organs by modulating the diameter of the vasculature [57,121,122]. This function likely occurs via directly activating the MT_1_ and MT_2_ receptors located on endothelial and vascular smooth muscle cells, and indirectly by its antioxidant properties to effect vascular tone. MT_1_ and MT_2_ receptors have been localized to a variety of arterial beds in humans, including the aorta, and coronary and cerebral arteries. Interestingly, melatonin receptors MT_1_ and MT_2_ are not expressed in all blood vessels so melatonin only exerts these potent vasoactive effects in specific regions of the vasculature [57]. The role of melatonin in the cardiovascular system at large has been summarized elsewhere and will not be covered in detail in this review [123].

Evidence that melatonin modifies production of NO is, unsurprisingly, vascular bed specific. In porcine coronary vascular smooth muscle, melatonin inhibited NO-induced increases in cGMP and artery relaxation via the MT_2_ receptor [124]. Interestingly, conflicting findings also revealed that melatonin exerts neuroprotective effects by suppressing NO production and enhancing activity of the endogenous antioxidant superoxide dismutase following oxidative injury [125,126]. Melatonin also increases NO availability, thereby inducing vasodilation of the mesenteric artery of healthy [127,128] and hypertensive rats [129].

These findings also translated to studies in human blood flow and vascular distribution. After human ingestion, melatonin reduced renal blood flow velocity and vascular conductance, enhanced forearm blood flow and vascular conductance, while not changing middle cerebral artery blood flow [121]. The decrease in renal blood flow could be eliminated by α-adrenergic receptor antagonism, indicating that melatonin is augmenting sympathetic outflow to the kidney. The effect on forearm blood flow, and lack of effect on middle cerebral artery blood flow was hypothesised to be due to a difference in melatonin receptor expression. An earlier study also demonstrated a lack of effect after a bolus injection of melatonin on cerebral blood flow, as measured in the basilar artery [130]. Once again, these studies support the concept that the relative distribution of the melatonin receptors influences the vascular effects of melatonin. Furthermore, in patients with three-vessel coronary disease, one month of oral melatonin treatment resulted in decreased plasma levels of VCAM and ICAM, while increasing plasma NO levels, providing further evidence that melatonin can protect against endothelial dysfunction [131].

In addition to increasing NO availability via MT_1_/MT_2_ receptors in endothelial cells, melatonin activates large conductance Ca^2+^-activated K^+^ (BK_Ca_) channels of smooth muscle cells, the role of which is in relaxation [128]. However, as the effects of melatonin are highly variable depending on the vasculature, the mechanisms explored in the mesenteric artery may not be translated to other vascular beds. It is hypothesised that melatonin, via its receptors, changes calcium and potassium channel regulation, and may also directly activate guanylate cyclase [132]. This is supported by an in vitro study that demonstrated melatonin-induced relaxation was only partially inhibited when NOS, the enzyme responsible for NO production, and guanylate cyclase were blocked [133]. In this chronic NO inhibited rat model of hypertension, melatonin treatment for 5 days significantly reduced basal mean arterial pressure. These rats had been administered the NO synthase (NOS) blocker L-NAME for 14 days, indicating that a mechanism beyond NO must be responsible for the observed antihypertensive effect of melatonin [113]. Though the conflicting nature of data regarding vasoactive effects of melatonin in animal vasculature limits the applicability to human populations, it certainly offers proof of concept that melatonin has the potential to directly modulate the vasculature. These mechanistic explorations may explain the hypotensive effect observed in humans [47,57,104,105,106].

Recently, a small open-label study sought to assess whether melatonin may allow safe prolongation of pregnancy in women with early-onset (<34 weeks gestation) preeclampsia by improving maternal endothelial dysfunction [47]. The administration of 30 mg daily of melatonin to 20 women with early-onset preeclampsia was associated with a six day increase in diagnosis to delivery interval and a reduced need for escalation of antihypertensive therapy. Consistent with those clinical outcomes, melatonin reduced placental and endothelial dysfunction [47]. Certainly this small study was open to bias, with small numbers, lack of blinding and use of historical controls. However, it was the first report of the use of melatonin therapeutically in preeclampsia. The correlation between both clinical and biochemical outcomes also supports a possible role for melatonin in restraining the otherwise unbridled placental and vascular oxidative stress of preeclampsia. While promising, these findings now require confirmation by a large randomised controlled trial before melatonin can be recommended for clinical use [134].

Pending further clinical trial assessment, melatonin has a number of attributes that would make it a particularly attractive therapy for preeclampsia (Figure 1). The safety of melatonin in pregnancy is well documented. It readily crosses the human placenta [135] and, even in high doses it has few, if any, adverse effects. Numerous animal studies have shown no maternal or fetal adverse effects from exogenous administration of melatonin [99,113,136]. Pilot studies of melatonin for women with growth restricted pregnancies (8 mg daily) [137], preeclampsia (30 mg daily) [47], and in 160 women undergoing in vitro fertilisation (8–16 mg daily) did not identify adverse outcomes from melatonin [138]. In a long-term contraceptive trial, women who took 75 mg melatonin a day for months reported no adverse effects [139] further supporting the safety profile of melatonin. The beneficial effects of melatonin for preeclampsia may extend beyond the mother. Current trials are investigating the potential for melatonin to protect the fetus in hostile intrauterine environments such as intra uterine growth restriction [103]. Research is underway to assess whether melatonin may offer benefit in augmenting induction of labour [54], preventing blood loss after caesarean section [140] and post-partum haemorrhage (IRCT2015050919037N9). In humans, melatonin also has anticonvulsant activity [141], which may be useful in the primary prevention of eclampsia.

## 5. Beyond Melatonin: Other Antioxidants for Preeclampsia

Melatonin is not alone in holding potential therapeutic utility as an antioxidant for the management of preeclampsia. Indeed, much excitement surrounded antioxidants upon the discovery that oxidative stress is likely a key player in the pathogenesis of the disease. This abated somewhat when the results from large clinical trials of Vitamin C and E were negative [142,143]. However, these therapies act only to directly scavenge ROS so do not carry the same potency as Nrf2-activators which upregulate a plethora of antioxidant enzymes and utilise innate cellular processes of signal amplification. Promising in vitro data [144,145] for esomeprazole saw it was rapidly evaluated in a clinical trial to delay delivery in women with preeclampsia [146]. That the clinical trial did not confirm any benefit of esomeprazole in diagnosed preeclampsia, despite preclinical observations, is a timely reminder of the need for thorough clinical trial assessments of new therapies. Potential benefit for high-risk women when given esomeprazole in the first trimester is under investigation. Resveratrol, found in red wine, is an inducer of the Nrf2 antioxidant pathway and has been shown to improve the health of trophoblasts and vascular cells in vitro [49,64,147]. Clinically, resveratrol improves the antihypertensive efficacy of nifedipine in managing preeclampsia [148]. A formal clinical trial of resveratrol would certainly be worthwhile. Another promising therapeutic candidate that addresses oxidative stress pathways is sulforaphane, a naturally occurring Nrf2 inducer found in cruciferous vegetables, particularly broccoli seed. Sulforaphane has been shown to have similar actions to melatonin in syncytiotrophoblast mitochondria, improving the resilience of the electron transport chain to both the hypoxic and oxidative mechanisms of injury of preeclampsia [94]. Sulforaphane directly reduces placental production of antiangiogenic proteins that trigger vascular dysfunction in preeclampsia [48]. As well as reducing inflammation and oxidative stress in vascular cells [20], sulforaphane protects omental blood vessels taken from pregnant women at the time of caesarean section against “preeclamptic-like” injury [63]. Not only does sulforaphane reduce sensitivity to vasoconstrictors, it also improves vasorelaxation in these injured blood vessels and, in supraphysiologic doses, can act as a direct vasodilator [63]. Clinical investigations of sulforaphane, as a broccoli extract formula, to treat established preeclampsia are underway and the results of that are eagerly awaited [149]. If the dose-finding studies are any indicator, sulforaphane looks very promising indeed. Even as a single dose, sulforaphane transiently reduced diastolic blood pressure and circulating sFlt1 levels in women with pregnancy hypertension at term [150].

## 6. Conclusions

Though essential in reducing stroke risk, antihypertensive therapy has reached the limit of its utility in the management of preeclampsia. Instead, we must now put to use our increased understanding of the pathogenesis of the disease to guide rational drug therapy. Specifically, antioxidants to mitigate the oxidative stress that underpins preeclampsia are likely an appropriate solution to protect the placenta and maternal endothelium. Melatonin is a safe, endogenous hormone with impressive antioxidant and antihypertensive effects that may make it a useful adjuvant for the management of preeclampsia. These antioxidant effects have been elucidated in placenta cells, in mitochondria, and in vascular cells, all key contributors to the pathogenesis of preeclampsia. The vasoactive effects of melatonin appear to be vessel specific in animals, though in humans it appears hypotensive and may reduce maternal blood pressure such that the inevitable delivery of the fetus can be safely delayed. Perhaps most excitingly, melatonin may offer benefits that extend beyond maternal care to protect the fetal brain in hostile intrauterine environments, such as FGR. Melatonin has been investigated for a host of other disorders related to pregnancy (summarized in Table 1). None of these studies raised safety concerns, even in high doses. Collectively, these findings highlight that melatonin is an exciting candidate for an adjuvant therapy for preeclampsia. Preliminary clinical trial evidence is promising and further, more fulsome, clinical evaluation is certainly warranted. An abundance of in vitro, animal-based and clinical evidence supports a role for melatonin in the management of preeclampsia, and indeed other disorders of pregnancy. Only through ongoing investigation of naturally occurring antioxidants, such as melatonin, can we hope to safely prolong pregnancy in severe preeclampsia and perhaps, for the first time in fifty years, offer a way to improve, and save, the lives of pregnant women with preeclampsia and their babies.

## Figures and Tables

**Table 1:** Summary of clinical trials investigating melatonin for pregnancy outcomes.

	Number of Participants	Intervention	Primary Outcome	Study Type
Swarnamani, K. et al., 2020 [54]	774 women undergoing induction of labour	Four doses of 10 mg of melatonin or placebo	Requirement for caesarean section	Double-blind randomised placebo-controlled trial
Palmer K. R. et al., 2019 [103]	336 women with FGR pregnancy between 23 + 0 and 31 + 6 weeks	30 mg per day or placebo	Neurodevelopment (difference of 5 points in the cognitive domain of the Bayley-III)	Triple-blind, parallel, randomised placebo-controlled trial
Khezri, M. et al., 2019 [140]	One hundred and twenty women undergoing caesarean section	3 or 6 mg of melatonin or placebo 20 min before caesarean section	Change in haemoglobin level	Double-blind randomised trial
Fernando, S. et al., 2018 [138]	One hundred and sixty women undergoing their first cycle of IVF or ICSI	2, 4 or 8 mg of melatonin or placebo twice daily	Clinical pregnancy rate	Double-blind randomised placebo-controlled trial
Hobson S.R. et al., 2016 [47]	Twenty women with preeclampsia	10 mg of melatonin three times daily	Diagnosis to delivery interval	Single-arm, open-label study
Alers N.O. et al., 2013 [137]	12 women with FGR pregnancies < 34 wks	4 mg melatonin twice daily	Biomarkers placental and circulating oxidative stress	Single-arm, open-label study
